# Antifungal activity of the piroctone olamine in experimental intra-abdominal candidiasis

**DOI:** 10.1186/s40064-016-2130-8

**Published:** 2016-04-16

**Authors:** Fabíola Maria Marques do Couto, Silene Carneiro do Nascimento, Silvio Francisco Pereira Júnior, Vanessa Karina Alves da Silva, André Ferraz Goiana Leal, Rejane Pereira Neves

**Affiliations:** Department of Mycology, Federal University of Pernambuco (UFPE), Av. da Engenharia, s/n, Cidade Universitária, Recife, PE CEP 50740-550 Brazil; Department of Antibiotics, Federal University of Pernambuco (UFPE), Av. da Engenharia, s/n, Cidade Universitária, Recife, PE CEP 50740-550 Brazil

**Keywords:** Activity antifungal, Piroctone olamine, Intra-abdominal candidiasis

## Abstract

This work aimed to evaluate the antifungal activity of piroctone olamine in the treatment of intra-abdominal candidiasis in an experimental model using Swiss mice. The mice (n = 6) were infected by intraperitoneal injection of 0.2 ml of *C. albicans* (10^7^cells/ml in saline). The animals were observed daily for clinical signs and mortality for 14 days. The treatment with piroctone olamine (0.5 mg/kg) was performed 72 h after infection by intraperitoneal administration. For comparison, a group of animals (n = 6) was treated with amphotericin B (0.5 mg/kg). The mycological diagnosis was made by collecting the liver, spleen and kidneys. Data regarding the fungal growth and mortality were analyzed statistically by Student’s *t* test and analysis of variance (ANOVA), with level of significance set at *P* < 0.05. The difference in fungal growth scoring between the control group and the treatment groups (piroctone olamine and amphotericin B) was statistically significant (*P* < 0.05). The difference in fungal growth scoring between the treatment groups (piroctone olamine and amphotericin B) was not statistically significant (*P* < 0.05).

## Background

Piroctone olamine (1-hydroxy-4-methyl-6-(2,4,4-trimethyl)-2-(1H)pyridinone,2-aminoethanol salt) is an ethanolamine salt of the hydroxamic acid derivative piroctone. This antifungal compound was first synthesized in 1979 by Schwarzkopf-Henkel (Germany) (Kim et al. 2011). It is a component of many cosmetic products such as anti-dandruff shampoo and hair rinses for scaly and irritated skin. One reason for scaling and irritation of the scalp is the colonization with *Malassezia* spp. and other yeasts. These microorganisms split parts of the sebum of the capillitium lipolytically into free fatty acids. These free fatty acids and microbial peroxidases lead to irritation of the skin and via an increase of mitosis to scaling (Sigle et al. 2006). Thus the piroctone olamine (PO) present in the shampoo and hair rinses have the ability to reduce microbial colonization. Its mechanism of action is complex and not completely understood. It is known that this agent has the ability to penetrate the cell membrane and form complexes with irons (Fe^2+^ e Fe^3+^), inhibiting energy metabolism in mitochondria of pathogenic fungi (Kim et al. 2011; Dubini et al. 2005).

Fungi can infect virtually any organ or structure in the abdomen. Intra-abdominal fungal infections can be divided in two groups according to their clinical presentation: localized, following surgery, trauma, or placement of foreign device; and disseminated, in critical ill or immunocompromised patients. It is important to recognize these infections early since serious complications, such as intestinal obstruction, bleeding, perforation, peritonitis, sepsis and death, can occur (Rebolledo and Sarria 2013). Among fungal agents that can cause intra-abdominal infection, *Candida* species are the most common. The antifungal drugs most commonly used to treat this type of invasive mycosis are amphotericin B, fluconazole and echinocandins (Bassetti et al. 2013).

The low number of available antifungal agents and the increased reports of yeasts resistant to conventional drugs can further complicate the treatment of intra-abdominal fungal infections (Quintero 2010; Yu et al. 2011). This work aimed to evaluate the antifungal activity of piroctone olamine in the treatment of intra-abdominal candidiasis in an experimental model using Swiss mice.

## Results and discussion

The in vitro antifungal activity of the drugs tested against isolates of *Candida* can be seen in Table 1. All *Candida* strains showed low minimum inhibitory concentrations (MICs) for PO (0.125–0.5 μg/mL) and amphotericin B (AMB) (0.03–1 μg/mL). However, the isolates were less susceptible to fluconazole (FLZ), for which the MICs ranged from 0.5 to 64 μg/mL.

**Table 1:** Evaluation of minimum inhibitory concentration (MIC) of fluconazole (FLZ), amphotericin B (AMB) and piroctone olamine (PO) against *Candida* species as agents of fungemia

Species	Number (%)	Drugs	MIC range (μg/ml)/average
*Candida albicans*	10 (23.26 %)	AMB	0.03–0.5 (0.01)
		FLZ	0.5–16 (4.6)
		PO	0.125–0.25 (0.19)
*C. parapsilosis*	13 (30.23 %)	AMB	0.03–0.5 (0.10)
		FLZ	0.5–64 (6.5)
		PO	0.125–0.5 (0.24)
*C. tropicalis*	09 (20.93 %)	AMB	0.03–0.06 (0.03)
		FLZ	0.125–64 (7.77)
		PO	0.125–0.5 (0.87)
*C. glabrata*	02 (4.65 %)	AMB	0.03–0.03 (0.03)
		FLZ	0.5–0.5 (0.5)
		PO	0.125–0.125 (0.12)
*C. guilliermondii*	07 (16.28 %)	AMB	0.03–1.0 (0.24)
		FLZ	2.0–4.0 (2.85)
		PO	0.125–0.25 (0.18)
*C. krusei*	02 (4.65 %)	AMB	0.03–1.0 (0.51)
		FLZ	16–32 (24)
		PO	0.125–0.25 (0.19)

The in vivo antifungal activity of PO and AMB in the experimental intra-abdominal candidiasis can be seen in Table 2. In the first stage of the experiment, the in vivo mortality rate was six dead animals in the untreated group and three dead animals in the groups treated with PO and AMB. This evaluation took place 14 days after infection with yeast. In the second stage of the experiment, the experimental model was repeated and the animals were sacrificed after 7 days of treatment. In the untreated group it was possible to isolate the fungus from the liver (all animals), spleen (three animals) and kidneys (three animals). In the animals treated with PO, the yeast was isolated from the liver (three animals) and spleen (two animals). In the group treated with AMB, the fungus was isolated from the liver (three animals) and spleen (one animal). The difference in FG scoring between the control group and the treatment groups (PO and AMB) was statistically significant (*P* < 0.05). The difference in FG between the treatment groups (PO and AMB) was not statistically significant (*P* < 0.05).

**Table 2:** Clinical and mycological evaluation of the antifungal activity of the piroctone olamine in experimental intra-abdominal candidiasis

Control group versus treatment group			
Rat/number	FG scoring		
	Liver	Spleen	Kidneys
*Control*			
1	1	0	1
2	1	1	1
3	1	0	1
4	1	1	0
5	1	1	0
6	1	0	0
*PO*			
1	0	1	0
2	0	1	0
3	0	0	0
4	1	0	0
5	1	0	0
6	1	0	0
*AMB*			
1	0	0	0
2	1	0	0
3	0	0	0
4	1	1	0
5	0	0	0
6	1	0	0

*PO* piroctone olamine, *AMB* amphotericin B, *FG* fungal growth in culture media: *(0)* no fungal growth and *(1)* fungal growth

The antimicrobial compound piroctone olamine is known to be effective in vitro against pathogenic fungi such as *Candida* species, *Aspergillus fumigatus* and dermatophytes. In addition to this antifungal activity, the drug also has good activity against gram-positive and gram-negative bacteria. Studies with mice, rats and dogs have shown that the PO has low toxicity. Research with animals has not shown any chromosomal abnormalities or acute pharmacological effects on the central nervous, cardiovascular or reproductive system or on other specific metabolic functions (Kim et al. 2011; Allgood et al. 1991).

Although the chemical properties of PO have been well characterized, no in vivo test of antifungal activity using models of invasive mycoses has been published. Invasive candidiasis is a frequent and life-threatening complication in critically ill surgical patients (Tissot et al. 2013). According to Tissot et al. 2013, some 30–40 % of episodes of recurrent gastrointestinal tract perforation or acute necrotizing pancreatitis are complicated by intra-abdominal candidiasis. Some clinical studies have reported that the morbidity and mortality of intra-abdominal candidiasis vary from 52 to 63 % (Tissot et al. 2013; Dupont et al. 2002). Treatment of this type of infection is a major challenge because of the difficulty of diagnosis, limited number of antifungals and emergence of strains resistant to the available drugs (Quintero 2010; Yu et al. 2011). In the in vitro susceptibility testing, we found that the clinical isolates of *Candida* spp. were less sensitive to FLZ.

## Conclusions

The results of this study indicate that PO has good antifungal activity in vitro and in vivo against clinical isolates of *Candida* spp. in an experimental model using Swiss mice involving treatment of intra-abdominal candidiasis. Future studies are planned to evaluate the antifungal activity of PO in other experimental models of invasive fungal infections, as well as the combination of this drug with other commercially available antifungal agents.

## Methods

### Antifungal activity of PO in vitro against *Candida* clinical isolates

A total of 43 clinical isolates of *Candida* spp. used in this study were provided by the URM Culture Collection of Federal University of Pernambuco (Table 1). All strains were isolated from invasive infections and they were preserved under mineral oil (Sherf 1943).

The susceptibility testing followed the broth microdilution method, in accordance with the standards published in Document M27-A3 from the Clinical and Laboratory Standards Institute. For quality control, the strains *C. parapsilosis* ATCC 22019 and *C. krusei* ATCC 6528 were included as quality control, as recommended by (CLSI 2008).

Amphotericin B (AMB) and fluconazole (FLZ) were used in the study as antifungal standards and the culture medium used was RPMI 1640 (Roswell Park Memorial Institute, Sigma Chemical Co., St. Louis, MO) with l-glutamine, 2.0 g/l glucose without sodium bicarbonate, buffered with 3-(N-morpholino) propanesulfonic acid (MOPS). The medium was sterilized by membrane filtration, 0.22 µm (Millipore, Darmstadt, Germany).

Ten different concentrations were used, ranging from 0.03 to 16 μg/mL of AMB and 0.125 to 64 μg/mL of FLZ. Piroctone olamine (Octopirox^®^, Sigma) was diluted in dimethyl sulfoxide (DMSO) to a stock solution concentration of 1600 μg/mL. The concentrations of piroctone olamine (PO) ranged from 0.0625 to 32 μg/mL.

The plates were incubated at 37 °C and readings were taken after 24 and 48 h of incubation. Two control wells, free from other fungi and yeasts, were included in the assay. The readings were made visually for comparison against the growth in control wells.

The minimum inhibitory concentration (MIC) was the lowest concentration capable of inhibiting visible growth of the isolates tested against the respective control well. Assays were performed in duplicate.

For FLZ, isolates with MIC < 8 μg/mL were considered susceptible, while isolates with MIC of 16–32 μg/mL were considered to have dose-dependent susceptibility (DDS) and MICs > 64 μg/mL were considered resistant. For AMB, isolates with MICs > 1.0 μg/mL were considered resistant. Guidelines for PO have not been established by reference documents.

### Antifungal activity of PO in an experimental model of intra-abdominal candidiasis

Adult male albino Swiss mice, weighing approximately 30 g, were obtained from the Department of Antibiotics, Federal University of Pernambuco (UFPE). The animals were treated in accordance with the established experimental procedures after approval by the institution’s ethics committee (protocol no. 23076.050932/2011-86). We used the immunosuppression protocol from Medeiros et al. 2010. A single injection of 0.5 mg/kg of dexamethasone (Teuto, Brazil) was administered intraperitoneally for three consecutive days and then at four-day intervals for the rest of the experiment.

The experiment was performed in two steps: (1) in the first, the treated and untreated animals were observed for mortality for 14 days, (2) in the second stage, the animals were sacrificed after 7 days of treatment for the mycological diagnosis. Seven days after initiation of immunosuppression, the mice (n = 6) were infected by intraperitoneal injection of 0.2 ml of *C. albicans* (10^7^cells/mL in saline). The concentration of *C. albicans* was adjusted using a spectrophotometer (Thermo Scientific Genesys™ 10S UV–Vis). The animals were observed daily for clinical signs and mortality for 14 days. The treatment with PO (0.5 mg/Kg) was performed 72 h after infection by intraperitoneal administration. For comparison, a group of animals (n = 6) was treated with amphotericin B (0.5 mg/Kg). The control group (n = 6) received only the solvent DMSO. The animals were euthanized in a CO_2_ chamber.

The mycological diagnosis was made by collecting the liver, spleen and kidneys. The organs were fragmented into pieces and inoculated onto culture medium contained in Petri dishes. The culture medium used was Sabouraud dextrose agar (SDA) (Difco) supplemented with chloramphenicol (50 mg/mL). The plates were incubated at 37 °C for up to 72 h. Grown yeast colonies were purified, isolated and identified through morphological and physiological characteristics (Barnett et al. 1983) to confirm the species involved in the infection. To evaluate the therapeutic efficacy of PO, fungal growth (FG) in culture media was scored according Leal et al. 2015 as follows: 0—no fungal growth in culture medium; and 1—fungal growth in culture medium. Part of the animal tissues collected was used for direct mycological examination. The samples were fragmented with a surgical scalpel and stained with 20 % potassium hydroxide (KOH).

Data regarding the FG and mortality were analyzed statistically by Student’s *t*-test and analysis of variance (ANOVA), with level of significance set at *P* < 0.05.

## References

[CR1] Allgood GS, Miller JM, Schardein JL (1991). The effect of piroctone olamine on reproduction of male and female rats. Fundam Appl Toxicol.

[CR2] Barnett JA, Payne RW, Yarrow D (1983). Yeasts: characteristics and identification.

[CR3] Bassetti M, Marchetti M, Chakrabarti A, Colizza S, Montero JG, Kett DH (2013). A research agenda on the management of intra-abdominal candidiasis: results from a consensus of multinational experts. Intensive Care Med.

[CR4] Clinical and Laboratory Standards Intitute (CLSI) (2008). Reference method for broth dilution testing of yeasts: approved standard-third edition M27-A3.

[CR5] Dubini F, Bellotti MG, Frangi A, Monti D, Saccomani L (2005). In vitro antimycotic activity and nail permeation models of a piroctone olamine (octopirox) containing transungual water soluble technology. Arzneimittelforschung.

[CR6] Dupont H, Paugam-Burtz C, Muller-Serieys C, Fierobe L, Chosidow D, Marmuse JP (2002). Predictive factors of mortality due to polymicrobial peritonitis with *Candida* isolation in peritoneal fluid in critically ill patients. Arch Surg.

[CR7] Kim Y, Alpmann P, Blaum-Feder S, Krämer S, Endo T, Lu D, Carson D, Schmidt-Wolf IGH (2011). Increased in vivo efficacy of lenalidomide by addition of piroctone olamine. In vivo.

[CR8] Leal AFG, Leite MC, Medeiros CSQ, Cavalcanti IMF, Wanderley AG, Magalhães NSS, Neves RP (2015). Antifungal activity of liposomal itraconazole formulation in experimental *Aspergillus flavus* keratitis with endophthalmitis. Mycopathologia.

[CR9] Medeiros CS, Pontes-Filho NT, Camara CA, Lima-Filho JV, Oliveira PC, Lemos SA, Leal AFG, Brandão JOC, Neves RP (2010). Antifungal activity of the naphthoquinone beta lapachone against disseminated infection whith *Cryptococcus neoformans var. neoformans* in dexamethasone imunossuppressed Swiss mice. Braz J Med Biol Res.

[CR10] Quintero CHG (2010). Resistencia de levaduras del género *Candida* al fluconazol. Infectio.

[CR11] Rebolledo M, Sarria J (2013). Intra-abdominal fungal infections. Curr Opin Infect Dis.

[CR12] Sherf AF (1943). A method for maintaining *Phytomonas sebedonica* in culture for long periods without transfer. Phytopathology.

[CR13] Sigle HC, Schafer-Korting M, Korting HC, Hube B, Niewerth M (2006). In vitro investigations on the mode of action of the hydroxypyridone antimycotics rilopirox and piroctone on *Candida albicans*. Mycoses.

[CR14] Tissot F, Lamoth F, Hauser PM, Orasch C, Fluckiger U, Siegemund M (2013). β-Glucan antigenemia anticipates diagnosis of blood culture-negative intraabdominal candidiasis. Am J Respir Crit Med.

[CR15] Yu L, Ling G, Deng X, Jin J, Jin Q, Guo N (2011). *In vitro* Interaction between fluconazole and triclosan against clinical isolates of fluconazole-resistant *Candida albicans* determined by different methods. Antimicrob Agents Chemother.

